# A Dynamic TDMA Scheduling Strategy for MANETs Based on Service Priority

**DOI:** 10.3390/s20247218

**Published:** 2020-12-16

**Authors:** Yufeng Ye, Xiangyin Zhang, Lanfeng Xie, Kaiyu Qin

**Affiliations:** 1 School of Aeronautics and Astronautics, University of Electronic Science and Technology of China, Chengdu 611731, China; yeyufeng@uestc.edu.cn (Y.Y.); kyqin@uestc.edu.cn (K.Q.); 2 Aircraft Swarm Intelligent Sensing and Cooperative Control Key Laboratory of Sichuan Province, Chengdu 611731, China; 3 AVIC Chengdu Aircraft Design and Research Institute, Chengdu 610091, China; J20Axlf@outlook.com

**Keywords:** dynamic distributed TDMA scheme, frame structure optimization, mobile ad hoc network (MANET), service priority, time division multiple access (TDMA)

## Abstract

Physical resource allocation strategy is a key factor affecting the performance of a mobile ad hoc network (MANET), which serves as a network model widely used in the sensor and detection field. For various perceived service information, it is significant for the allocation strategy to adapt to the dynamic service requirements and prioritize resource access for the service information with high priority to guarantee its real-time performance. In this paper, a novel dynamic time division multiple access (TDMA) scheduling strategy is proposed for MANETs. Firstly, a service priority-based dynamic TDMA scheduling (SP-DS) algorithm is presented, which introduces the service priority as a reference factor for slot allocation and fully considers the transmission throughput and end-to-end delay performance. Moreover, for improving the slot use of the whole system, a modified distributed color constraint heuristic (MD-CCH) scheme is presented to optimize the frame structure. The SP-DS and MD-CCH algorithms are combined to form the novel strategy. Simulation results have demonstrated that the proposed strategy has better performance in the slot use, slot allocation efficiency, end-to-end delay and transmission throughput compared with the existing slot allocation algorithms.

## 1. Introduction

### 1.1. Background and Motivation

The mobile ad hoc network (MANET) is a typical wireless distributed network, which is adapted to the requirements of communication and networking in the high dynamic and large spatial scale scenarios [1,2]. In contrast to the traditional cellular networks (GSM, LTE-A, 5th-Generation, etc.) [3], it has the characteristics of decentralization, infrastructure-less, self-organization, flexible networking and dynamic topology [4,5], which have made it be recognized as a promising network model that is widely used in the sensor and detection field [1,6,7], such as Internet of Things (IoT) [8], wireless sensor network (WSN) [9], unmanned aerial vehicle (UAV) swarm detection network [10] and tactical unmanned vehicle sensing system (UVSS) [11,12]. In these scenarios, with the limited network resources, various sensor nodes interact the perceived information with each other in a distributed multi-hop cooperative transmission manner [13]. To improve the network adaptability and quality of service (QoS), a reasonable resource scheduling strategy is needed for optimizing network resource use and resource allocation efficiency [14,15], which highlights the importance of the physical resource allocation strategy [16]. The traditional physical resource allocation strategy includes two main categories: centralized and distributed [14,17]. The centralized strategy has a central controller to collect the information of the entire network and generate a resource allocation scheme for each node. Its distribution result is close to optimal but the overall network information is needed for each allocation, which results in poor flexibility and survivability, and not suitable for the network with dynamic topology [18]. For improving the topological adaptability, the distributed allocation strategy is proposed. The mainstream scheme adopted by the distributed physical resource allocation strategy is distributed time division multiple access (TDMA) slot allocation scheme [19,20], which can be basically divided into two categories: (a) Static distributed TDMA scheme and (b) Dynamic distributed TDMA scheme [14,21,22].

The static distributed TDMA scheme is a fixed time slot allocation scheme without contention, which assigns the same fixed time slot to each node in the network. It aims at guaranteeing the fairness of resource allocation. However, nodes in the MANET scenario usually have different service requirements so that the static distributed TDMA scheme is unable to make full use of time slot resources and cannot adapt to the dynamic service requirements [21].

To realize the dynamic allocation of resources among different nodes according to the various needs of each node, the dynamic distributed TDMA scheme has been proposed. In the dynamic scheme, each node selects the time slot according to its local (two-hop range) environment information and generates an allocation strategy in a distributed manner, which has good scalability and flexibility so that can adapt to the dynamic service requirements of different nodes [23,24]. These characteristics make the dynamic distributed TDMA scheme show better applicability than the static distributed TDMA scheme in the MANET scenario. Researchers have recognized the dynamic distributed TDMA scheme as the future development trend and proposed various related algorithms.

### 1.2. Related Work

In the five-phase reservation protocol (FPRP) [25], network nodes perform a five-phase dialogue mechanism to reserve time slots. When a reservation conflict occurs, each related node within two-hop range will give up its reservation and wait for a backoff time. This protocol can resolve data transmission conflicts, but it takes a long allocation time. The distributed randomized (DRAND) time slot scheduling algorithm [26] adopts a completely random coin tossing mechanism to limit the probability of nodes sending slot reservation requests in each control frame. This scheme will pick up a successful node in each round. However, it has no clear reference factors for slot reservation, which results in the high collision rate and low allocation efficiency. The localized DRAND (L-DRAND) algorithm [27] divides the whole sensor network into various areas. Each local area will select a relay node. To achieve efficient data delivery, the priority of time slot allocation in L-DRAND algorithm is set as follows: the relay node of each area owns the highest priority and the node closer to the relay node in each local area is assigned in priority. This scheme improves the slot allocation efficiency but it introduces a large amount of distance information measured by sensors, which increases the network energy consumption. According to the DRAND, Li et al. [17] proposed the distributed TDMA slot scheduling algorithm based on energy-topology factor (E-T-DRAND). An energy topology (E-T) factor is defined according to the residual energy and one-hop neighbors of a node. The priority of the nodes to send time slot reservation request is determined by E-T factor. Compared with DRAND algorithm, this scheme improves the energy use and time slot allocation efficiency of the whole network. According to the E-T-DRAND, Li et al. [18] further proposed the distributed TDMA scheduling algorithm based on exponential backoff rule and energy-topology factor (EB-ET-DRAND). This scheme introduces an exponential backoff mechanism, i.e., the backoff time of a node is set according to the number of rejected messages it receives. It maximizes the amount of useful information in the network and further improves the slot allocation efficiency and energy use.

The above dynamic distributed TDMA scheduling algorithms have resolved resource allocation conflicts from different perspectives. However, they do not fully consider time slot multiplexing when designing frame structure. Typically, only one node is arranged in each time slot and the number of time slots in each TDMA frame (i.e., frame length) [28] is set to be the total number of network nodes, which results in a long frame length and low time slot use. In this case, considering the reuse of time slots, Bryan et al. [29] proposed various kinds of frame structure optimized slot scheduling algorithm based on the graph coloring theory. Firstly, a centralized coloring algorithm based on the color constraint heuristic (CCH) factor, namely centralized CCH slot assignment (CSA-CCH) algorithm [29] is presented. It uses the CCH factor as the reference for coloring order and the result is close to optimal. However, it introduces the topology information of the entire network, which is not suitable for a MANET. A distributed color constraint heuristic slot assignment (DSA-CCH) algorithm [29] is proposed for the distributed networks. Its coloring order is determined by the ratio of the weight sum of the colored nodes to the weight sum of the total nodes within two-hop range. When this ratio exceeds a threshold, a node starts to color itself. The simulations show that DSA-CCH algorithm optimizes the frame length while improving slot use. Yao et al. [30] introduced the Lexicographic Max-Min (LMM) criterion into the rate allocation problem. They defined a slot reuse control parameter ε in the LMM rate allocation problem and proposed a TDMA scheduling algorithm by iteratively calculating ε. This algorithm achieves the goal of reusing slots, and optimizes the frame length and throughput of the sensor network. Chang et al. [31] proposed a novel TDMA protocol, which establishes a linear programming model, and adopts the simulated annealing and particle swarm optimization algorithm to solve this model. A slot allocation scheme with good slot use was obtained. This protocol optimizes the frame length and data transmission delay, but it does not completely guarantee the continuity of information transmission among different nodes.

For the existing dynamic distributed TDMA schemes, all the transmission information is default to the same service priority. In practical applications, the sensing information often has different service priorities and QoS requirements [32]. It is necessary to prioritize resource access for the sensing information with high service priority and guaranteed its real-time performance through the dynamic resource allocation strategy [33]. Moreover, as various service information (i.e., sensing information) perceived by the sensor nodes needs be transmitted from the source node to the destination node in time, the end-to-end transmission delay of different service information should be reduced as much as possible to improve the throughput of the whole network system [34,35]. In addition, the problem of frame structure optimization is not fully considered in most scheduling algorithms. Therefore, the dynamic distributed TDMA scheduling strategy still needs to be further researched.

### 1.3. Innovations and Contributions

In this paper, a service priority-based dynamic TDMA scheduling (SP-DS) algorithm for MANETs system is proposed. The SP-DS algorithm firstly introduces the service priority as a reference factor in the distributed time slot allocation. Meanwhile, it performs the random token generation mechanism to solve the reservation conflicts of the nodes with same priority. Then, based on the consideration of multi-hop cooperative characteristic in the end-to-end transmission of MANETs [36,37,38], the source node and routing nodes have been jointly considered as a whole link when allocating slots. In SP-DS algorithm, a transmission path from source node to the destination node is firstly constructed through the routing module in the reservation phase. After that, the order of time slot reservations among different nodes is made consistent with the order of nodes on the transmission path to ensure the continuity of service information transmission and reduce the end-to-end transmission delay. Furthermore, an adaptive time slot allocation mechanism based on binary tree model is adopted in SP-DS algorithm according to the data size and real-time requirements of different service priority information. In addition, we propose a distributed vertex coloring-based frame structure optimization algorithm, called the modified distributed color constraint heuristic (MD-CCH) algorithm, to optimize frame length and improve the time slot use. Finally, we perform extensive simulations using Network Simulator NS-3.27 [39] on Ubuntu to compare our scheduling strategy with the existing time slot allocation algorithms. Simulation results have shown that our scheduling strategy can process the perceived important service information in priority. Moreover, it has better performance in the slot use, slot allocation efficiency, end-to-end transmission delay and data transmission throughput.

**Figure 1:**
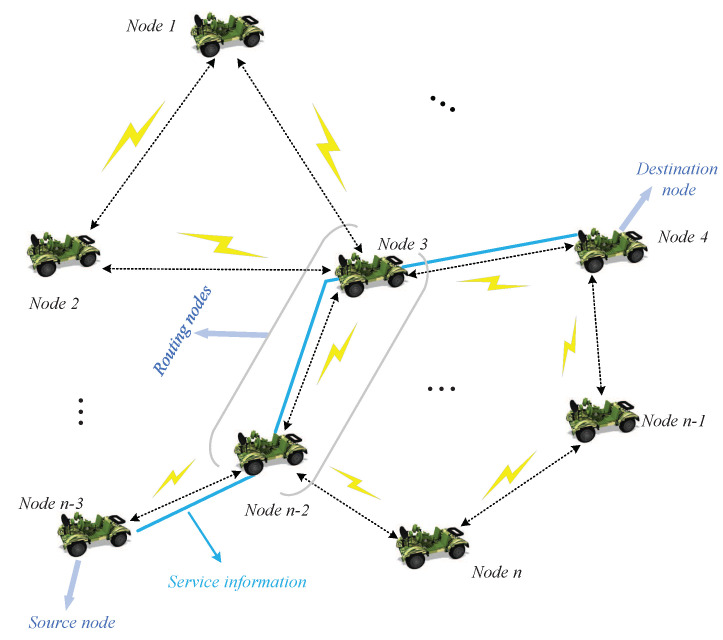
The application scenario of tactical unmanned vehicle sensing system (UVSS).

In this paper, we focus on the dynamic TDMA scheduling strategy based on service priority for MANETs. The application scenario of the proposed strategy is shown in Figure 1. In this figure, the tactical unmanned vehicle sensing system (UVSS) is taken as an example, each node is embodied as a unmanned vehicle equipped with intelligent sensors. The whole system is a distributed network with random and dynamic topology composed of wireless mobile nodes. With no central controller in the network, all nodes have both routing and terminal functions. The service information perceived by the source node is forwarded to the destination node through multi-hop routing in a distributed manner.

The main contributions of this paper are as follows:

- A novel service priority-based distributed broadcast (SP-DB) mechanism is proposed, which introduces the service priority factor to prioritize various sensing information and enhance the adaptability to dynamic service requirements.
- A time slot reservation mechanism based on the consideration of multi-hop cooperative characteristic in the end-to-end transmission of MANETs is presented to ensure the continuity of service information transmission.
- A binary tree model-based adaptive time slot allocation mechanism is adopted to handle the traffic load of different service priority information efficiently.
- The MD-CCH algorithm for frame structure optimization is proposed to improve slot use of the system and further reduce end-to-end delay.

Rest parts of this paper are organized as follows: Section 2 describes the proposed SP-DS algorithm. The MD-CCH algorithm is presented in Section 3. Section 4 evaluates the impact of priority on the service information and compares the performance of proposed TDMA scheduling strategy with the existing slot allocation algorithms. Section 5 concludes the paper.

## 2. The Service Priority Based Dynamic TDMA Scheduling Algorithm

In this section, we propose a service priority-based dynamic TDMA scheduling (SP-DS) algorithm. Firstly, we describe the service priority-based distributed broadcast mechanism. Then, the time slot reservation mechanism is presented and we analyze the mathematical expectation of reservation rounds that a node needs to pass for acquiring time slot in SP-DS algorithm. Finally, the binary tree model-based adaptive time slot allocation mechanism is proposed.

### 2.1. The Service Priority Based Distributed Broadcast Mechanism

In the initial phase of network, each node has a predefined service information table that contains different types of services and the corresponding priority numbers. The service with a larger priority number has a higher priority level, as shown in Table 1.

**Figure 2:**
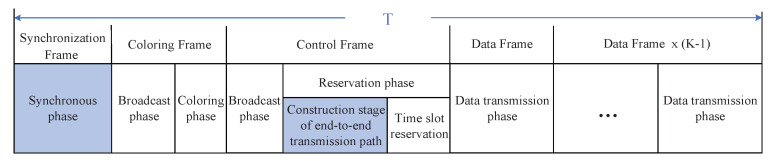
The superframe structure.

The superframe T for performing the dynamic TDMA scheduling strategy is shown in Figure 2. It is composed of a synchronization frame, a coloring frame, a control frame, and *K* data frames. *K* is determined by the clock deviation. Within the allowable range of clock deviation, *K* can be appropriately increased [18]. The superframe will continue to circulate in the entire time domain. The synchronization frame is used to achieve the time synchronization of all nodes. This paper focuses on the TDMA scheduling strategy, so the synchronization algorithm is not discussed here, as with reference [17,18]. The coloring frame is used to perform the frame structure optimization algorithm, which will be discussed in Section 3. The SP-DS algorithm is executed in the control frame and data frames are responsible for data transmission.

**Figure 3:**
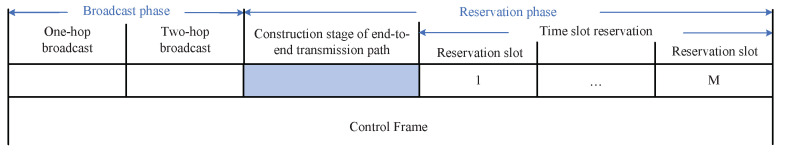
The control frame structure.

As shown in Figure 3, a control frame consists of a broadcast phase and a reservation phase. The broadcast phase is further divided into a one-hop broadcast stage and a two-hop broadcast stage.

In the one-hop broadcast stage, all nodes broadcast *Hello-packets* to their one-hop neighbors. A *Hello-packet* contains node ID, token (only for nodes with reservation requirements and the details will be mentioned later) and priority number (only for nodes with reservation requirements). In the two-hop broadcast stage, each node that receives the *Hello-packets*, will forward them to its one-hop neighbors. As a result, each *Hello-packet* can be received by all neighbors within the two-hop range of a sender node.

At the end of the broadcast phase, the nodes with reservation requirements will compare the captured priority information with their own. Only the nodes with the highest priority number have the opportunity to enter the reservation phase. The nodes with lower service priority have to stay in the state of IDLE and wait for a backoff time. According to reference [17,18], the SP-DS algorithm divides the state transition process of nodes into IDLE, REQUEST, GRANT, and RELEASE. The backoff time is set to be 3 times of the transmission delay [17].

We stipulate that each node with reservation requirement needs to perform a random token generation mechanism at the beginning of control frame in each round, i.e., a random integer between 0 and 255 will be generated as a token. This mechanism is shown in Algorithm 1 (refer to line 4). The token will be broadcast in a *Hello-packet*. When there are multiple nodes with the same highest priority number within two-hop range, the node with the smallest token will enter the reservation phase. At the beginning of control frame in the next round, tokens for all nodes with reservation requirements will be randomly regenerated. This scheme is to guarantee the fairness of multiple nodes with the same service priority when they are making time slot reservations. Furthermore, it can ensure that only one node within the two-hop range attempts to send a reservation request in a reservation slot of reservation phase.
**Algorithm 1** The SP-DS Time Slot Allocation Algorithm of Node *j*1:$state_{j}$: the four states of node *j*;2:**The broadcast process of node *j*:**3:**if** ( node *j* has a reservation requirement) **then**4:    $j_{token}$ = rand()%255;5:    Generate a *Hello-packet* that contains node ID, token and the priority number;6:**else**7:    Generate a *Hello-packet* that contains node ID;8:**end if**9:Broadcast j’s own *Hello-packet*;10:Forward the received *Hello-packets*;11:**The reservation process of node *j*:**12:**if** ( node *j* success in competition) **then**13:    Construct a transmission path through the routing module and calculate *S*;14:    $state_{j}$= REQUEST;15:    Broadcast the reservation request to its one-hop neighbors;16:    **if** ( node *j* receives the grant messages from all one-hop neighbors ) **then**17:        Select the corresponding transmission slot DS${}^{i}$;18:        Broadcast release message;19:        $state_{j}$= RELEASE;20:    **else**21:        Broadcast failure message;22:        $state_{j}$= IDLE;23:        wait for back_off time;24:    **end if**25:**else**26:    $state_{j}$= IDLE;27:    wait for back_off time;28:**end if**29:**Node *n* is one of *j***’***s* one-hop neighbors, and receives the request from *j*:**30:**if** ( $state_{n}$= IDLE or $state_{n}$= RELEASE ) **then**31:    $state_{n}$= GRANT;32:    Send a grant to node *j*;33:**else**34:    Send a reject to node *j*;35:**end if**36:**Node *n* is one of *j***’***s* neighbors within two-hop range, and receives the release or failure from *j*:**37:forward the received messages to n’s one-hop neighbors;

### 2.2. The Time Slot Reservation Mechanism

After finishing the broadcast phase, the nodes with the highest priority level and the smallest token within their two-hop range will enter the reservation phase. These nodes are called reservation nodes in this paper. The reservation phase includes a construction stage of end-to-end transmission path and a time slot reservation stage, as shown in Figure 3. The time slot reservation stage is further divided into *M* reservation slots according to the maximum number of nodes within two-hop range of the whole network. *M* can be calculated by the MD-CCH algorithm, which will be discussed in Section 3.2.

In the construction stage of end-to-end transmission path, each reservation node will build a transmission path from itself to the destination node through the routing module, so as to know its next hop node ID in advance before starting time slot reservation. After the transmission path is constructed, all nodes on that path can get their next hop node IDs.

**Figure 4:**
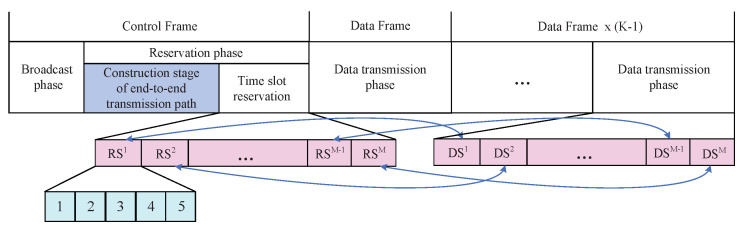
The corresponding relationship between reservation slot and data transmission slot.

In the time slot reservation stage, each reservation slot RS${}^{i}$ is corresponding to a data transmission slot DS${}^{i}$, as shown in Figure 4. If a node needs to use a certain slot DS${}^{i}$, it must make a reservation in the corresponding slot RS${}^{i}$. Only when the reservation is successful, can that node perform data transmission in the corresponding DS${}^{i}$ time slots in the subsequent *K* data frames. Each time reservation slot RS${}^{i}$ is further divided into 5 stages.

In the first stage of RS${}^{i}$, each reservation node will broadcast a slot reservation request to its one-hop neighbors. In the second stage, the nodes receiving the reservation request will send a grant or reject message to the source node. A source node is considered to successfully reserve a time slot DS${}^{i}$ only when it receives the grant messages from all its one-hop neighbors. In the third stage, if the reservation is successful, the source node will broadcast a success message, its priority number, and the node ID with the highest reservation priority within its two-hop range in the next slot RS${}^{i+1}$ to its one-hop neighbors, all of which are included in the release message mentioned in Algorithm 1 (refer to line 18). Otherwise, it will only broadcast a failure message. In the fourth stage, the one-hop neighbors of source node will forward the received messages to their one-hop neighbors. In the fifth stage, the two-hop neighbors of source node will forward the received messages to their one-hop neighbors. The node that received its own ID in the third stage of RS${}^{i}$ must send a reservation request in the first stage of RS${}^{i+1}$ on the condition that its received node IDs in the fourth and fifth stage of RS${}^{i}$ are not within its two-hop range or the received priority numbers are lower than its own.

In this way, the neighbors within the two-hop range of both the reservation node and its next hop node are informed of the reservation status of slot DS${}^{i}$ and the node ID with the highest reservation priority in the next slot RS${}^{i+1}$.

**Figure 5:**
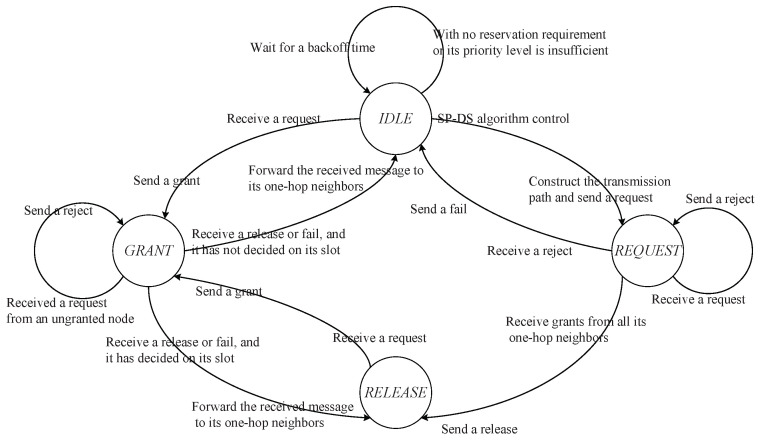
The state diagram of the SP-DS algorithm implementation.

**Figure 6:**
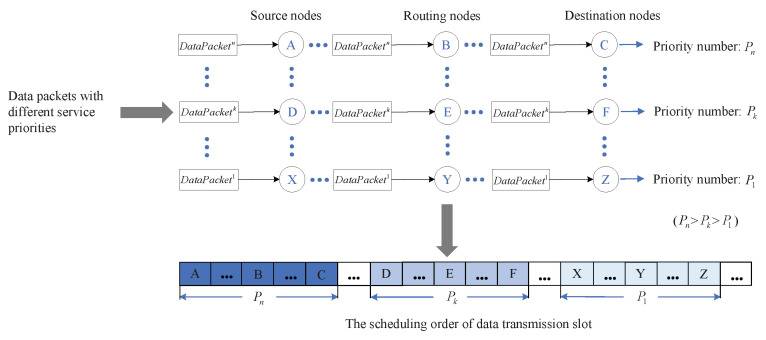
Transmission paths of different service priority information and data transmission slot allocation order of corresponding nodes.

Figure 5 and Table 2 show the state variation process of nodes in the implementation of SP-DS algorithm. It is necessary to point out that a node with a successful time slot reservation knows the node ID with the highest reservation priority within its two-hop range in the next reservation slot RS${}^{i+1}$ is due to the following reasons:

- In the first stage of RS${}^{i}$, each reservation node will calculate the number of slots *S* that can be continuously reserved in the current control frame according to the binary tree model-based adaptive slot allocation mechanism, which will be mentioned in Section 2.4. When the reserved slots do not reach *S*, a reservation node needs to continue to reserve in RS${}^{i+1}$, so it will broadcast its own ID in the third stage of RS${}^{i}$.
- A reservation node has constructed a transmission path from itself to the destination node through the routing module before it starts time slot reservation, so it knows its next hop node ID when it is reserving time slot. To ensure that the current service information can be continuously transmitted by the routing nodes on the transmission path (as shown in Figure 6) to reduce the unnecessary delay, the order of time slot reservations among different nodes needs to be kept consistent with the order of routing nodes on the transmission path. In this case, the reservation node will broadcast its next hop node ID in the third stage of RS${}^{i}$ on the condition that the number of slots it has reserved reaches *S*. The node that received its own ID in the third stage of RS${}^{i}$ must send a reservation request in the first stage of RS${}^{i+1}$, provided that its received node IDs in the fourth and fifth stage of RS${}^{i}$ are not within its two-hop range or the received priority numbers are lower than its own. Otherwise, it will back off. This is to ensure as far as possible that the next hop node of reservation node can reserve the slot DS${}^{i+1}$ successfully so that it can continue to transmit the same service information.

When the broadcast phase is completed, all nodes have collected the ID information of their neighbors within two-hop range. Therefore, when the node ID with the highest reservation priority in slot RS${}^{k+1}$ broadcasted by a node $N_{a}$ in the fourth or fifth stage of slot RS${}^{k}$ exceeds the two-hop range of a node $N_{b}$, $N_{b}$ can send a reservation request in the first stage of slot RS${}^{k+1}$ on the condition that its priority number is the largest within its two-hop range while its token is the smallest among the nodes with same priority number.

### 2.3. The Mathematical Expectation of Reservation Rounds

If *j*-node has a need to reserve time slot, then C(j,k) is a set that in the *k*-th round, the contenders within the two-hop range that have slot reservation requirements and the same priority number as *j*-node. In each round, which node of all the competitors (include *j*-node) can apply for time slot is determined by their randomly generated tokens. Therefore, the probability of these nodes acquiring time slot is equal in each round.

L(j,k) denotes that *j*-node gets the right to reserve time slot in the *k*-th round. Pr(*j*${}_{leave}$, *t*) represents the probability that *j*-node acquires time slot in the *t*-th round (t≥1), *t* has a geometric distribution. Pr(*j*${}_{leave}$, *t*) is bounded as follows:

(1) $$\begin{array}{cc} \mathrm{Pr}(j_{leave},t) & \geq \mathrm{Pr}(L\left(j_{leave},1\right)) \end{array}$$

(2) $$\begin{array}{cc} \; & =\mathrm{Pr}\left(L\left(j,1\right)\right)\prod_{i\in C(j,1)}\left(1-\mathrm{Pr}(L(i,1))\right) \end{array}$$

(3) $$\begin{array}{cc} \; & =\frac{1}{M_{2}+1}\prod_{i\in C(j,1)}\left(1-\mathrm{Pr}(L(i,1))\right) \end{array}$$

(4) $$\begin{array}{cc} \; & =\frac{1}{M_{2}+1}\prod_{i\in C(j,1)}\left(1-\frac{1}{\left|C(i,1)\right|+1}\right) \end{array}$$

(5) $$\begin{array}{cc} \; & =\frac{1}{M_{2}+1}{\left(1-\frac{1}{\left|C(i,1)\right|+1}\right)}^{\left|C(j,1)\right|} \end{array}$$

(6) $$\begin{array}{cc} \; & \geq \frac{1}{M_{2}+1}{\left(1-\frac{1}{\left|C(j,1)\right|+1}\right)}^{\left|C(j,1)\right|} \end{array}$$

(7) $$\begin{array}{cc} \; & >\frac{1}{M_{2}+1}\cdot \left(\frac{1}{e}\right) \end{array}$$

In Equation (3), *M*${}_{2}$ represents the number of contenders with the same priority number as *j*-node within the two-hop range. Let δ be the number of total neighbors for *j*-node within its two-hop range. It is clear that *M*${}_{2}$≤δ, the equality holds only when the priority numbers of all the nodes within the two-hop range of *j*-node are the same.

The transformation from Formulas (4) to (6) holds because the probability of contender *i* acquires time slot in the first round is Pr(L(i,1)), as shown in Equation (8). Since *i*-node’s contenders include *j*-node, according to reference [26], we have that ${\left(\left|C(i,1)\;\right|+1\right)}_{min}=\left|C(j,1)\;\right|+1$.

(8) $$\begin{array}{c} \mathrm{Pr}\left(L\left(i,1\right)\right)=\frac{1}{\left|C(i,1)\right|+1} \end{array}$$

Equation (7) holds because we can derive Equation (10) from Equation (9). Equation (10) holds only when $\left|C(j,1)\right|$ approaches infinity, i.e., the number of nodes in the network is infinite.

(9) $$\begin{array}{c} \lim_{x\to \infty }{\left(1+\frac{1}{x}\right)}^{x}=e \end{array}$$

(10) $$\begin{array}{c} \lim_{\left|C(j,1)\right|\to \infty }{\left(1-\frac{1}{\left|C(j,1)\right|+1}\right)}^{\left|C(j,1)\right|}=\frac{1}{e} \end{array}$$

The probability of *j*-node acquires time slot in the *t*-th round (t≥1) has a lower bound $P_{low}$, as shown in Equations (11) and (12).

(11) $$\begin{array}{c} \mathrm{Pr}\left(t=k\right)=P_{low}{\left(1-P_{low}\right)}^{k-1}, \end{array}$$

where

(12) $$\begin{array}{c} P_{low}=\frac{1}{\left(M_{2}+1\right)}\cdot \frac{1}{e} \end{array}$$

In conclusion, we can obtain the upper bound on the mathematical expectation of reservation rounds that a node needs to pass for acquiring time slot as follows:

(13) $$\begin{array}{c} E\;\left[\;T\;\right]=\frac{1}{P_{low}}=\left(M_{2}+1\right)\cdot e \end{array}$$

### 2.4. The Binary Tree Model Based Adaptive Slot Allocation Mechanism

The sensing information with different service priorities has various requirements for transmission delay. The high service priority information asks for high level real time performance and needs to be transmitted in priority within the shortest delay. The lower service priority information could be moderately postponed. In this case, the nodes with higher service priorities can be allowed to continuously reserve multiple slots in a control frame. Meanwhile, it is also necessary to ensure as far as possible that the nodes with lower service priorities have slots to reserve and avoid the slot starvation of these nodes.

**Figure 7:**
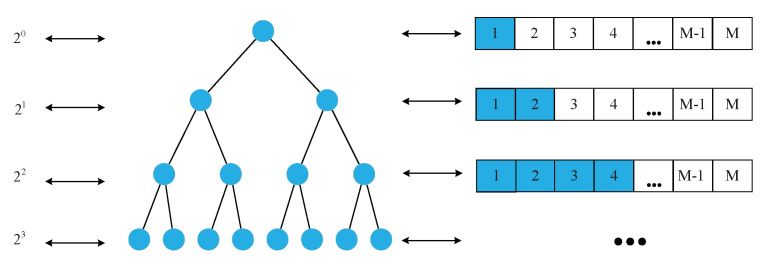
The mapping relationship between binary tree model and the number of reservable slots.

The SP-DS algorithm proposes a binary tree model-based adaptive time slot allocation mechanism, which maps the number of slots allocated to the nodes with different service priorities to the number of binary tree nodes of different levels, as shown in Figure 7. According to the predefined service information table (Table 1), we stipulate the number of slots that one node can continuously reserve in a control frame has a positive correlation with its corresponding priority number.

Based on Figure 7, the functional relationship between the number of slots $S^{{}^{\prime }}$ that a node can continuously reserve in a control frame and its priority number *N* can be initially expressed as

(14) $$\begin{array}{c} S^{{}^{\prime }}=2^{N} \end{array}$$

The node with the lowest priority number (zero) can only reserve one slot in each control frame. As *N* increases, $S^{{}^{\prime }}$ increases based on Equation (14). Considering the total number of available slots is limited, an upper limit constraint needs to be made on $S^{{}^{\prime }}$ in order to prevent the nodes with lower service priorities from slot starvation due to long waiting time.

As shown in Figure 4, the time slot reservation stage of control frame and the data transmission phase of data fame are respectively divided into *M* slots, where *M* represents the maximum number of nodes within the two-hop range of whole network. To ensure the quality of transmission delay of nodes with lower service priorities, the functional relationship between $S^{{}^{\prime }}$ and *N* is modified into Equation (15).

(15) $$\begin{array}{c} S^{{}^{\prime }}=min\left\{2^{N},\left\lceil \frac{M}{2}\right\rceil \right\} \end{array}$$

At the same time, since the service information of different data size asks for various total number of transmission slots, further optimization constraints need to be placed on the number of slots that a node can continuously reserve in a control frame.

Assume that the total data size of the service information that a node currently needs to transmit is $D_{sum}$, which will be updated after each round. The current transmission bandwidth is $V_{c}$ and the transmission slot length is $t_{slot}$. The total number of slots $\bar{S}$ required by a node to complete the transmission of its current service information can be expressed as

(16) $$\begin{array}{c} \bar{S}=\left\lceil \frac{D_{sum}}{V_{c}\cdot t_{slot}}\right\rceil \end{array}$$

Combining Equations (15) and (16), the number of slots *S* that a node should continuously reserve in the current control frame is expressed in Equation (17), where *K* is the number of data frames in each round.

(17) $$S=\left\{\begin{array}{cc} \; & S^{{}^{\prime }}\;,\;\;\;\bar{S}>K\cdot S^{{}^{\prime }}\\ \; & \left\lceil \frac{\bar{S}}{K}\;\right\rceil \;,\;\;\;\bar{S}\leq K\cdot S^{{}^{\prime }}\; \end{array}\right.$$

In DRAND [26], EB-ET-DRAND [18], and DSA-CCH [29] algorithms, a node can only reserve one slot in each control frame. Assume that the number of data frames in each round is uniformly set to *K*, the available transmission time $t_{trans}^{1}$ for a node adopting these algorithms in each round is

(18) $$\begin{array}{c} t_{trans}^{\;1}\;\;=\;\;K\cdot t_{slot} \end{array}$$

After adopting the adaptive slots allocation mechanism, as shown in Algorithm 2, the range of transmission time $t_{trans}^{2}$ available for a node in each round is

(19) $$\begin{array}{c} K\cdot 2^{0}\cdot t_{slot}\leq t_{trans}^{\;2}\leq K\cdot S\cdot t_{slot} \end{array}$$

It can be seen that this allocation mechanism makes the transmission slots available for nodes with higher service priority increase in each round, so that it can reduce the unnecessary transmission delay and improve the real time performance of high service priority information.
**Algorithm 2** The Binary Tree Model Based Adaptive Slot Allocation Mechanism1:Initialize *K*, *M*, *N*, $D_{sum}$, $V_{c}$, $t_{slot}$;2:$\bar{S}\leftarrow ceil\left(D_{sum}/(V_{c}*t_{slot})\right)$.3:$S^{{}^{\prime }}\leftarrow min\left(pow\left(2,N\right),ceil\left(M/2\right)\right)$.4:**if** ( $\bar{S}>K*S^{{}^{\prime }}$ ) **then**5:    $S=S^{{}^{\prime }}$;6:**else**7:    $S=ceil(\bar{S}/K)$;8:**end if**

## 3. The Distributed Vertex Coloring Based Frame Structure Optimization Algorithm

In this section, we discuss the details about the TDMA frame structure optimization algorithm. Firstly, the model of graph vertex coloring is presented. Then, the modified distributed color constraint heuristic (MD-CCH) algorithm is proposed.

### 3.1. The Model of Graph Vertex Coloring

The traditional frame structure of time slot allocation algorithms set the number of slots for TDMA schedule to the number of nodes allowed in the network. It defaults a slot can only be occupied by one node and does not consider time slot multiplexing, which introduces redundant slots and result in a low slot use.

In the process of time slot assignment, one slot can be simultaneously used by various nodes, provided that they are outside the two-hop range. According to the graph coloring theory: the adjacent vertices cannot use the same color, the optimization of TDMA frame structure can be modeled as a vertex coloring problem for a graph. Each color represents a time slot. The fewer colors used for graph coloring, the fewer time slots needed for TDMA schedule, which optimizes the frame length, improves slot use and further reduces the end-to-end delay of the system [29].

**Figure 8:**
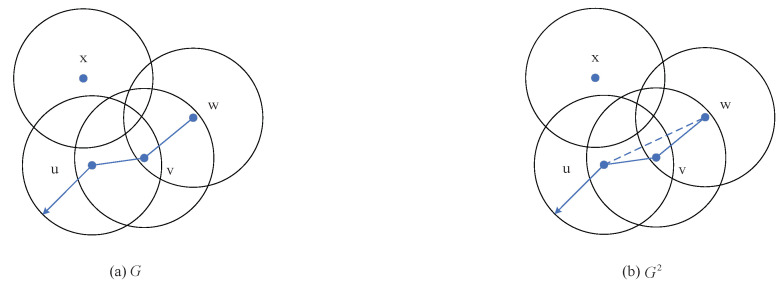
The unit circle set *G* and the square of graph *G* ($G^{2}$).

As shown in Figure 8a, we assume that each node has the same broadcast range. The communication area of a node is represented by a unit circle. In graph *G*, if nodes can directly communicate with each other, they are connected by solid edges. That is to say, only the nodes within one-hop range are connected in *G*. However, TDMA schedule needs to consider all nodes within two-hop range for avoiding the direct and hidden conflicts, so graph *G* cannot be directly used for coloring.

The square of graph *G* is graph $G^{2}$, as shown in Figure 8b. It can be seen that in addition to retaining the same topological structure connected by solid edges as in *G*, $G^{2}$ has an extra dotted edge for connecting node *u* and node *w*. The dotted edge (*u*, *w*) exits in $G^{2}$ only when the distance between *u* and *w* is 2 edges in *G*. Therefore, the nodes within two-hop range are connected in $G^{2}$, which allows $G^{2}$ be used for coloring. As stated in reference [40,41], the problem of coloring graph $G^{2}$ is equivalent to the distance-2 coloring problem, namely *L*(1, 1)-labeling problem. A reasonably designed coloring algorithm is needed to solve this problem for optimising TDMA frame structure.

### 3.2. The Modified Distributed Color Constraint Heuristic Algorithm

The DRAND algorithm [26] adopts a completely random order for coloring. As the number of nodes increases, the number of colors used may reach $\Delta (G^{2})$ in the worst case. The CSA-CCH scheme [29] is a centralized coloring algorithm, which needs to introduce the topology information of the entire network and is not suitable for MANETs. The DSA-CCH scheme [29] is a distributed coloring algorithm. However, it does not have a clear limit on the initial coloring nodes, which brings more uncertainties to the coloring effect as the number of nodes increases. In this case, we propose the modified distributed color constraint heuristic (MD-CCH) algorithm, which makes a restriction on the selection of initial coloring nodes.

The nodes with more neighbors have a higher collision probability during data transmission. They have fewer options when coloring, so that need to be prioritized. The conventional method is to color from the node with the largest degree. However, considering that the degree of a node in a square graph is consisted of its one-hop and two-hop neighbors, the one-hop neighbors have a more direct effect on a node during data transmission, so they should occupy a higher reference weight. In this paper, we introduce the $Node_{level}$ factor, which is consisted of a weight sum of the one-hop neighbors (NumberOneHops) and two-hop neighbors (NumberTwoHops), as shown in Equation (20).

(20) $$\begin{array}{c} Node_{level}=2\cdot \;NumberOneHops+NumberTwoHops \end{array}$$

**Figure 9:**
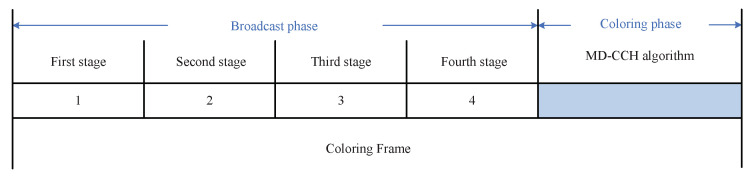
The coloring frame structure.

The MD-CCH algorithm is executed in the coloring frame, which is composed of a broadcast phase and a coloring phase. The broadcast phase is further divided into 4 stages, as shown in Figure 9.

In the first stage of broadcast phase, all nodes broadcast their own IDs to their one-hop neighbors. Based on the received messages, each node will create a table of ID information about its one-hop neighbors, namely the ID information table of one-hop neighbors, to calculate its own NumberOneHops. In the second stage, each node broadcasts its ID information table of one-hop neighbors labeled with its own ID to the one-hop neighbors. According to these tables, all nodes will create the ID information tables of their two-hop neighbors (used to calculate their own NumberTwoHops) and get the NumberOneHops of all their one-hop neighbors. In the third stage, each node firstly calculates its own $Node_{level}$ and then broadcasts it along with its own ID and the previously received ID information tables of one-hop neighbors to its one-hop neighbors. Based on these tables, all nodes can get the NumberOneHops of all their two-hop neighbors. The NumberOneHops information of all the one-hop and two-hop neighbors will be used to choose the starting node when there are multiple nodes with the same $Node_{level}$ within two-hop range. According to the received $Node_{level}$ and ID information, all nodes will create the $Node_{level}$ information tables of their one-hop neighbors. In the fourth stage, each node broadcasts the $Node_{level}$ information table of its one-hop neighbors to its one-hop neighbors. Based on these tables, all nodes will create the $Node_{level}$ information tables of their two-hop neighbors.

When the broadcast phase is completed, each node starts to perform the MD-CCH algorithm in the coloring phase. The detailed coloring rules of coloring phase are as follows:

- The coloring starts from the nodes with the highest value of $Node_{level}$ within their two-hop range. If there are multiple nodes with the same $Node_{level}$ within two-hop range, the node with more one-hop neighbors (NumberOneHops) will be chosen as the starting node.
- All the one-hop neighbors of the starting nodes are colored based on the descending order of their NumberOneHops.
- The remaining nodes decide when to start coloring according to the DSA-CCH algorithm. That is to say, a node starts to color itself when the color_score, as shown in Equation (21) [29], exceeds a threshold. The threshold is set through experimental simulation (in our experimental simulation, considering the balance of coloring order between breadth-first and depth-first, the threshold is set to be 0.28 for making the weight of coloring order towards a breadth-first [29]).

(21) $$\begin{array}{c} color\_score=\frac{2\cdot \;ColoredOneHop+ColoredTwoHop}{2\cdot \;NumberOneHops+NumberTwoHops} \end{array}$$

The MD-CCH algorithm is shown in Algorithm 3. Compared with CSA-CCH, MD-CCH retains the distributed coloring scheme to adapt to the distributed networks. Moreover, based on the DSA-CCH algorithm, it introduces the $Node_{level}$ factor as the selection reference of the initial coloring nodes through a 4-stage broadcast mechanism. The MD-CCH algorithm optimizes the uncertainties of coloring effect that is caused by the improper selection of the initial coloring nodes. Furthermore, the optimization effect of frame structure can be guaranteed.
**Algorithm 3** The MD-CCH Distributed Vertex Coloring Algorithm of Node *j***Input:**$Node_{level}$_set[]; NumberOneHops_set[]; Colors_set[].**Output:** Consumed colors (*M*) in Colors_set[].1:$Node_{level}$_set[]: The $Node_{level}$ information array of node *j* and its all neighbors within two-hop range;2:NumberOneHops_set[]: The NumberOneHops information array of node *j* and its all neighbors within two-hop range;3:Colors_set[]: The array of colors used for coloring each node;4:select_color(): Choose a color that is not overlapped with other neighbors within two-hop range to start coloring.5:**if** ((max($Node_{level}$_set[]) == j’s$Node_{level}$) && (max(NumberOneHops_set[](with the same $Node_{level}$ as *j* )) == j’sNumberOneHops) **then**6:    select_color(Colors_set[]);7:**else**8:      **if** ( *j* is one of the starting node’s one-hop neighbors and the starting node has finished coloring) **then**9:          **if** ( j’sNumberOneHops is the maximal among the starting node’s one-hop neighbors) **then**10:           select_color(Colors_set[]);11:        **else**12:           Listen and wait;13:        **end if**14:    **else**15:        **if** (2*(j’sColoredOneHop) + j’sColoredTwoHop)/(2*(j’sNumberOneHops) + j’sNumberTwoHops) ≥color_score **then**;16:           select_color(Colors_set[]);17:        **else**18:           Listen and wait;19:        **end if**20:    **end if**21:**end if**

## 4. Experiment and Performance Evaluation

In this section, we implement a MANET simulation system on NS-3 platform to evaluate the proposed dynamic TDMA scheduling strategy. The impact of priority on the performance of various service information is assessed. In addition, this strategy is compared with the existing algorithms from the aspects of slot use, slot allocation efficiency, end-to-end delay and throughput.

### 4.1. Experiment Settings

**Figure 10:**
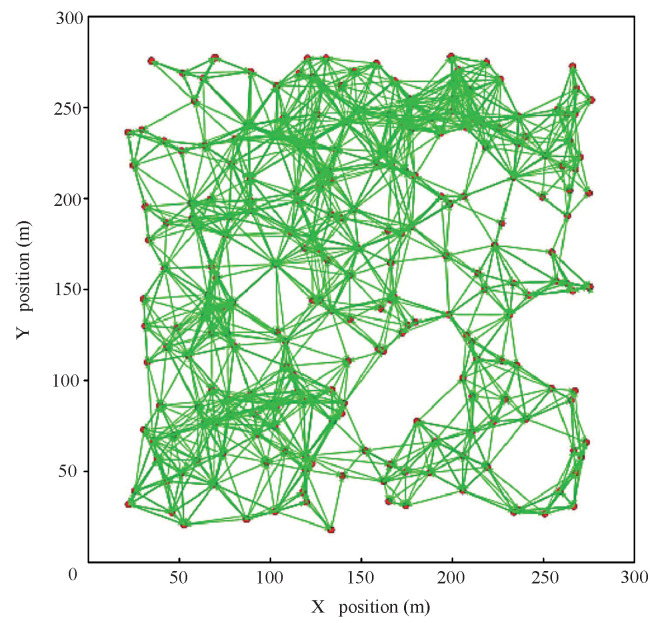
The random network topology on NS-3 simulation platform.

The NS-3.27 version is used to implement our simulations. This platform has the ability of abstract modeling and performance evaluation for the spatially dynamic distributed topological networks. The topology of the whole network is determined by the randomly scattering points in two-dimensional space, as shown in Figure 10.

The specific simulation parameters are shown in Table 3. To facilitate comparative analysis, we configure the basic simulation parameters based on the reference [26]. The broadcast communication range of each node is 40 m and the link capacity is 2 Mbps. The size of the movable area for all nodes is 300 m × 300 m.

The transport layer adopts user datagram protocol (UDP) instead of transmission control protocol (TCP), because UDP is a connectionless protocol that can more accurately reflect the packet reception and loss, thus better evaluating the throughput and delay performance of various algorithms. In the routing module, the ad-hoc on-demand distance vector routing (AODV) protocol is used to build the transmission path.

### 4.2. Experiment Results and Performance

For the proposed dynamic TDMA scheduling strategy, firstly, its validity in prioritized service information transmission is evaluated. Then, it is assessed by comparing with the existing DRAND, EB-ET-DRAND, and DSA-CCH algorithms from the aspects of slot use, slot allocation efficiency, end-to-end delay and throughput. All the data results are averaged after 10 repeat experiments, as with reference [18]. The detailed numerical simulations and performance analysis are as follows (the error bars in the graphs represent the confidence intervals with a confidence level of 0.95):

#### 4.2.1. Performance Comparison of Different Service Priority Information

In this section, we evaluate the impact of priority on the service information in terms of throughput and delay. The number of nodes is set to 50. The packet size in each service flow is set to 256 bytes and 1000 packets are sent per second. Two priority levels of service information are set, which are divided into the high service priority information (HS) and the low service priority information (LS). The number of flows in each simulation is set to an even number that satisfies the following condition:

(22) $$\begin{array}{c} flows_{\left(HS\right)}:flows_{\left(LS\right)}=1:1 \end{array}$$

**Figure 11:**
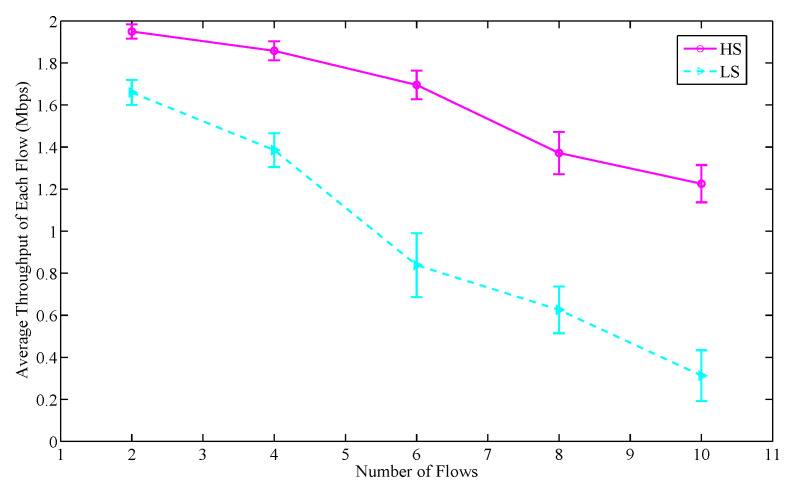
The throughput comparison of different service priority information.

**Figure 12:**
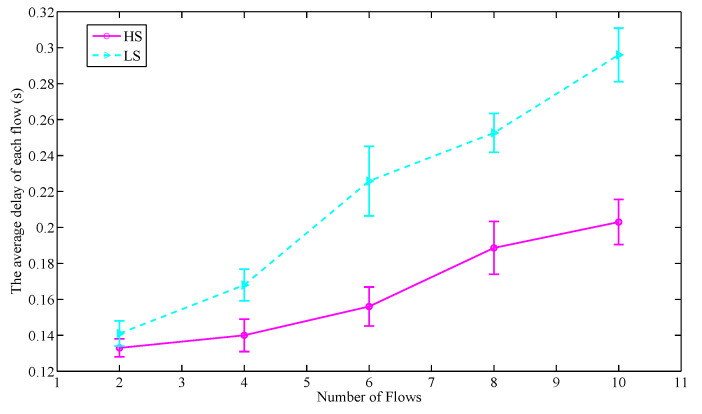
The delay comparison of different service priority information.

As shown in Figure 11 and Figure 12, with the increasing of flows, for the service information of two priority levels, the average throughput of each flow decreases while the average delay of each flow increases. The average throughput decline rate and delay growth rate of HS are lower than that of LS. This is because the DS-SP algorithm enables the nodes carrying HS to reserve time slot resources in priority, thereby prioritizing data transmission. At the same time, due to the data size of the two kinds of information is the same, the binary tree model-based adaptive slot allocation mechanism makes the nodes carrying HS have a higher upper limit on the number of slots that can be continuously reserved in each control frame. Therefore, the nodes carrying HS can get more slots in each round to transmit data, thus the improvement of their throughput and reduction of their unnecessary waiting delay is achieved. The results indicate that the throughput of each flow in LS is on average 43.9% lower than that of HS while the delay of each flow in LS is on average 30.1% higher than that of HS. That is to say, the proposed scheduling strategy can guarantee the performance of HS, thus achieving the goal of processing the perceived important service information in priority.

#### 4.2.2. Number of Colors

**Figure 13:**
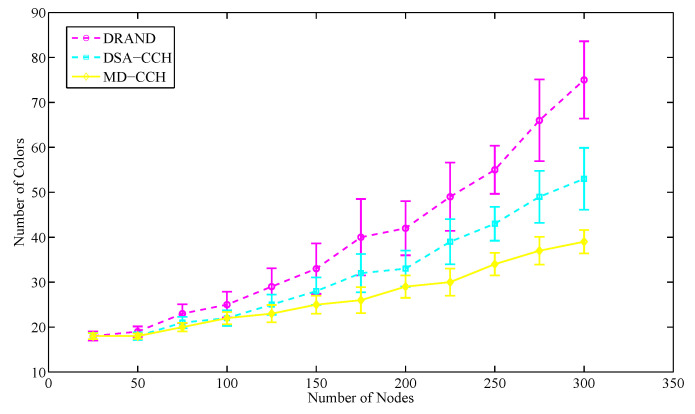
The average colors consumed in different size of network nodes.

The number of colors represents the corresponding number of time slots needed to be set in the frame structure for TDMA schedule. The fewer colors used, the shorter frame length and higher slot use we can get. Figure 13 shows the coloring effects of three different algorithms. Since EB-ET-DRAND algorithm does not consider the optimization of TDMA frame structure [18], it is not compared in this section. The number of network nodes varies from 25 to 300 and color_score is set to be 0.28 for making the weight of coloring order towards a breadth-first.

According to Figure 13, we can see that the number of colors increases with the increasing of nodes. When the nodes are less than 100, the coloring effect of DSA-CCH algorithm and MD-CCH algorithm is similar. As the number of nodes further increases, MD-CCH algorithm uses fewer colors than DSA-CCH algorithm because it strengthens the constraints on the initial coloring nodes. The number of colors consumed by the MD-CCH algorithm is on average 25.8% less than that of DRAND algorithm and on average 12.4% less than that of DSA-CCH algorithm. That is to say, the frame structure optimized by MD-CCH algorithm has the highest slot multiplexing rate. The results indicate our proposed scheduling strategy can efficiently improve slot use.

#### 4.2.3. Running Time and Number of Reservation Rounds

Running time denotes the time of successfully allocating slot resources for each node in the network. It is proportional to the number of reservation rounds. In this section, the network topology consists of 250 nodes placed randomly and the number of neighbor nodes within two-hop range varies from 4 to 70.

**Figure 14:**
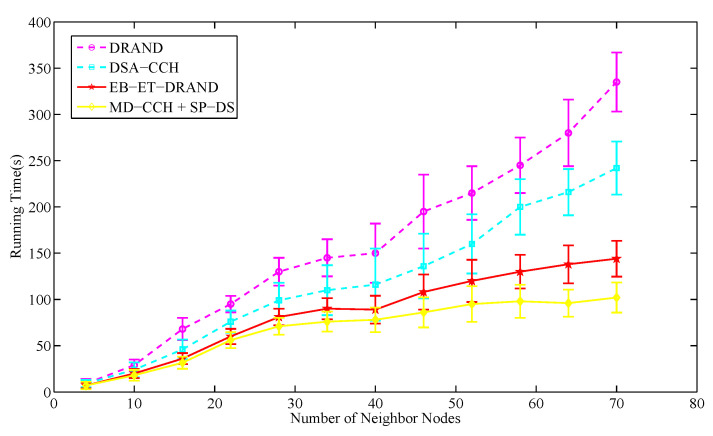
The average running time for successful allocating a time slot in different size of neighbor nodes.

**Figure 15:**
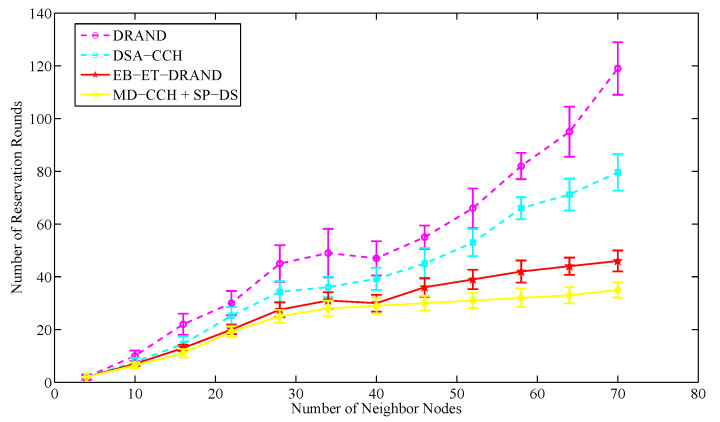
The average number of reservation rounds for successful allocating a time slot in different size of neighbor nodes.

Figure 14 and Figure 15 respectively show the average running time and average reservation rounds of various schemes in different size of neighbor nodes. It can be seen that with the increase of neighbor nodes, the running time and the number of reservation rounds of each scheme increase. Compared with EB-ET-DRAND algorithm and the novel strategy, DRAND and DSA-CCH algorithms have a higher growth rate. This is because they do not introduce a clear reference factor in the time slot allocation, which brings more reservation conflicts when allocating time slots. As a result, many nodes fail to make a reservation and need to resend their requests. With the further increase of neighbor nodes, this phenomenon will become more serious. Eventually, when the number of neighbor nodes exceeds 40, the growth rates of DRAND and DSA-CCH algorithms increase sharply, resulting in non-convergence.

The EB-ET-DRAND algorithm adopts an exponential backoff mechanism and introduces the energy information as a reference factor for time slot allocation. It can reduce the failure and collision probability of time slot allocation to some extent. When the neighbor nodes are less than 22, the running time and reservation rounds in the novel strategy are similar to that of EB-ET-DRAND algorithm. As the further increase of neighbor nodes, the novel strategy takes less allocation time. This is because its SP-DS algorithm introduces the service priority as a reference factor for slot allocation and adopts the random token generation mechanism to solve the reservation conflicts of the nodes with same priority. Compared with the EB-ET-DRAND algorithm, it can better ensure that only one node within two-hop range can enter the reservation phase to send a reservation request. The failure and collision probability of slot allocation is reduced to a greater extent. In addition, its MD-CCH algorithm has optimized the TDMA frame structure, which makes the frame length shorter than that of EB-ET-DRAND algorithm and further reduces the latency of its time slot allocation. The running time in the novel strategy is on average 50.3% less than that of DRAND algorithm and on average 16.2% less than that of EB-ET-DRAND algorithm. The results indicate the proposed time slot scheduling strategy is effective for improving slot allocation efficiency.

#### 4.2.4. End-to-end Delay and Throughput

The end-to-end delay means the average time taken for a packet to travel from the source node to the destination node, while the throughput represents the ratio of the amount of data successfully received by the destination node over a period of time to the transmission time. In this section, we arbitrarily select a priority level of service information and gradually increase its flows to compare the end-to-end delay and throughput variations of the whole system caused by transmitting this service information in different schemes. The packet size in each service flow is set to 256 bytes and 1000 packets are sent per second. The number of network nodes is set to 50.

**Figure 16:**
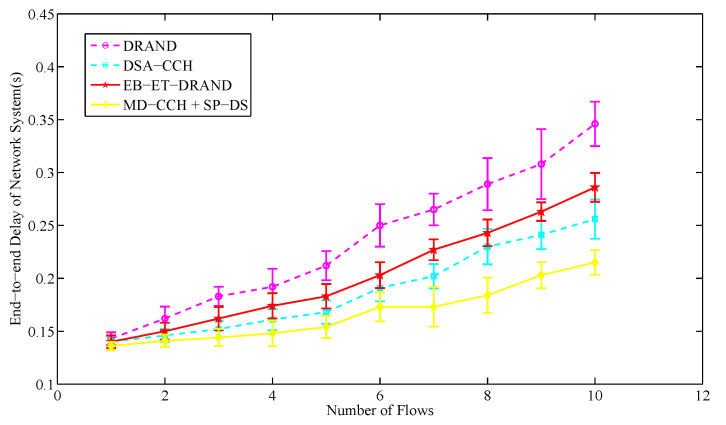
The average end-to-end delay of network system in different number of flows.

As we can see from Figure 16, the end-to-end delay of network system increases with the number of flows. With the same number of flows, the novel strategy has the lowest end-to-end delay. This is because in its SP-DS algorithm, the source node and routing nodes have been jointly considered as a whole link to determine the order of time slot reservation when allocating time slot, which fully guarantees the end-to-end transmission delay of service information. Moreover, the binary tree model-based adaptive allocation mechanism of its SP-DS algorithm allows the related nodes to continuously reserve multiple slots in each control frame, so that more slots can be obtained for data transmission in each round, thereby reducing the unnecessary waiting delay. In addition, its MD-CCH algorithm optimizes the frame length, which further reduces the end-to-end delay of information transmission among different nodes.

**Figure 17:**
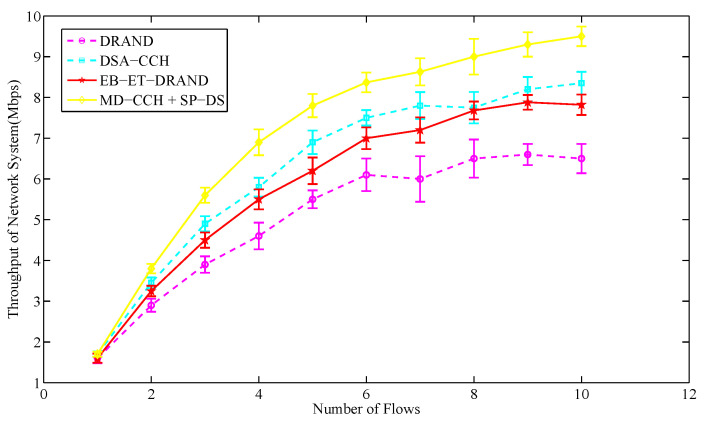
The average throughput of network system in different number of flows.

Figure 17 shows that as the number of flows increases, the throughput of network system increases. When the network throughput is close to saturation, the throughput of the novel strategy is the largest. This is because according to the previous description, with the sending rate unchanged, the novel strategy reduces the end-to-end delay of the whole network, which in turn makes the throughput of the network increase. The results show that the average end-to-end delay of the novel strategy is 26.3% lower than that of DRAND algorithm while its average network throughput is 37.9% higher than that of DRAND algorithm. That is to say, the proposed time slot scheduling strategy can well optimize the end-to-end delay and data throughput of the entire network.

## 5. Conclusions

The division of service priority has important application requirements in MANETs, whose performance is greatly affected by the dynamic distributed TDMA slot allocation scheme. It is necessary to prioritize resource access for the sensing information with high service priority and guaranteed its real-time performance as much as possible. In this paper, a novel dynamic TDMA scheduling strategy for MANETs based on service priority is proposed. This strategy includes two parts: time slot allocation and frame structure optimization. In the part of time slot allocation, the SP-DS algorithm is proposed. Firstly, in the broadcast mechanism of SP-DS algorithm, the service priority is introduced as a reference factor for slot allocation and a random token generation mechanism is performed to solve the reservation conflicts of the nodes with same priority. Then, based on the consideration of multi-hop cooperative characteristic in the end-to-end transmission of MANETs, a time slot reservation mechanism is presented to fully guarantee the end-to-end transmission delay of service information. Moreover, a binary tree model-based adaptive time slot allocation mechanism is adopted to handle the traffic load of different service priority information and ensure the real-time performance of high service priority information. In the part of frame structure optimization, the MD-CCH algorithm is proposed to optimize frame length for improving slot use, which further reduces the end-to-end delay of the whole network system. The simulation results demonstrate the validity of the proposed strategy in prioritized service information transmission. Furthermore, compared with several typical algorithms, our proposed TDMA scheduling strategy shows better performance in the slot use, slot allocation efficiency, end-to-end transmission delay and data transmission throughput. In the future research work, considering the dynamic topological characteristics of MANETs, we plan to optimize the time slot scheduling strategy for further improving the robustness of the transmission path.

## Figures and Tables

**Table 1:** The service information table.

Priority Number (N)	Service Type
0	A
1	B
2	C
3	D
4	E
...	...

**Table 2:** The state transition table of SP-DS algorithm.

NO.	Current State	Event	Action/Condition	Next State
1	IDLE	With the largest priority number within the two-hop range and the smallest token among the nodes of same level	Construct the transmission path and send a slot reservation request	REQUEST
2	IDLE	Receive a request from one of the one-hop neighbors	Send a grant	GRANT
3	IDLE	With no reservation requirement or its priority level is insufficient	Backoff	IDLE
4	REQUEST	The grant information from all one-hop neighbors is received	Send a release	RELEASE
5	REQUEST	Receive a request from another node	Send a reject	REQUEST
6	REQUEST	Receive a reject from one of the one-hop neighbors	Send a fail	IDLE
7	RELEASE	Receive a request from one of the one-hop neighbors	Send a grant	GRANT
8	GRANT	Receive a request from an ungranted node	Send a reject	GRANT
9	GRANT	Receive a release or fail, and it has not been allocated time slot	Forward the received message to its one-hop neighbors	IDLE
10	GRANT	Receive a release or fail, and it has been allocated time slot	Forward the received message to its one-hop neighbors	RELEASE

**Table 3:** Simulation parameters.

Parameter	Value
Topology model	ns3::Random Rectangle Position Allocator
Mobile model	ns3::Random Way Point Mobility Model
Propagation loss model	ns3::Log Distance Propagation Loss Model
Propagation delay model	ns3::Constant Speed Propagation Delay Model
Simulation area	300 m × 300 m
Movement speed of nodes	4 m/s
Number of nodes	[25,300]
Range of neighbour nodes	[4,70]
Link capacity	2 Mbps
Broadcasting range(m)	40
Packet size	256 Byte
Packet sending rate	1000 packets/s
Routing protocol	AODV
Transfer protocol	UDP
Number of flows	1–10
Data flow generator	OnOffApplication

## References

[1] Nadeem A., Howarth M.P. (2013). A survey of MANET intrusion detection & prevention approaches for network layer attacks. IEEE Commun. Surv. Tutor..

[2] Glass S., Mahgoub I., Rathod M. (2017). Leveraging MANET-based cooperative cache discovery techniques in VANETs: A survey and analysis. IEEE Commun. Surv. Tutor..

[3] Barik P.K., Shukla A., Datta R., Singhal C. (2020). A resource sharing scheme for intercell D2D communication in cellular networks: A repeated game theoretic approach. IEEE Trans. Veh. Technol..

[4] Ye Q., Zhuang W., Li L., Vigneron P. (2016). Traffic-load-adaptive medium access control for fully connected mobile ad hoc networks. IEEE Trans. Veh. Technol..

[5] Cho J., Swami A., Chen I. (2011). A survey on trust management for mobile ad hoc networks. IEEE Commun. Surv. Tutor..

[6] Luo J., Ye D., Xue L., Fan M. (2009). A survey of multicast routing protocols for mobile ad-hoc networks. IEEE Commun. Surv. Tutor..

[7] Peng M., Liu W., Wang T., Zeng Z. (2020). Relay selection joint consecutive packet routing scheme to improve performance for wake-up radio-enabled WSNs. Wirel. Commun. Mob. Comput..

[8] Ye Q., Zhuang W. (2017). Token-based adaptive MAC for a two-hop Internet-of-Things ennabled MANET. IEEE Internet Things J..

[9] Butun I., Morgera S.D., Sankar R. (2014). A survey of intrusion detection systems in wireless sensor networks. IEEE Commun. Surv. Tutor..

[10] Gupta L., Jain R., Vaszkun G. (2016). Survey of important issues in UAV communication networks. IEEE Commun. Surv. Tutor..

[11] Lee J.S., Yoo Y., Choi H.S., Kim T., Choi J.K. (2019). Energy-efficient TDMA scheduling for UVS tactical MANET. IEEE Commun. Lett..

[12] Kim D., Kim J., Ko Y. (2018). BiPi-TMAC: A bidirectional-pipelined TDMA for reliability and QoS support in tactical unmanned vehicle systems. IEEE Access.

[13] Lwin M.T., Yim J., Ko Y.-B. (2020). Blockchain-based lightweight trust management in mobile ad-hoc networks. Sensors.

[14] Zheng C., Huang S., Wei J., Dong Q. (2019). MD-MAC: A distributed TDMA protocol based on desynchronization for multi-hop topologies. Sensors.

[15] Natkaniec M., Kosek-Szott K., Szott S., Bianchi G. (2013). A survey of medium access mechanisms for providing QoS in ad-hoc networks. IEEE Commun. Surv. Tutor..

[16] Roh B., Han M., Hoh M., Kim K., Roh B. Tactical MANET architecture for unmanned autonomous maneuver network. Proceedings of the 2016 IEEE Military Communications Conference (MILCOM).

[17] Li Y., Zhang X., Zeng J., Wan Y., Ma F. (2017). A distributed TDMA scheduling algorithm based on energy-topology factor in internet of things. IEEE Access.

[18] Li Y., Zhang X., Qiu T., Zeng J., Hu P. (2017). A distributed TDMA scheduling algorithm based on exponential backoff rule and energy-topology factor in internet of things. IEEE Access.

[19] Lin C., Zadorozhny V., Krishnamurthy P., Park H., Lee C. (2011). A distributed and scalable time slot allocation protocol for wireless sensor networks. IEEE Trans. Mob. Comput..

[20] Long J., Dong M., Ota K., Liu A. (2017). A green TDMA scheduling algorithm for prolonging lifetime in wireless sensor networks. IEEE Syst. J..

[21] Suriyachai P., Roedig U., Scott A. (2012). A survey of MAC protocols for mission-critical applications in wireless sensor networks. IEEE Commun. Surv. Tutor..

[22] Alvi A.N., Bouk S.H., Ahmed S.H., Yaqub M.A., Sarkar M., Song H. (2016). BEST-MAC: Bitmap-assisted efficient and scalable TDMA-based WSN MAC protocol for smart cities. IEEE Access.

[23] Diaz-Anadon M.O., Leung K.K. (2011). TDMA scheduling for event-triggered data aggregation in irregular wireless sensor networks. Comput. Commun..

[24] Gholami E., Rahmani A.M., Dehghan Takht Fooladi M. (2015). Adaptive and distributed TDMA scheduling protocol for wireless sensor networks. Wireless Pers. Commun..

[25] Zhu C., Corson M.S. (2001). A five-phase reservation protocol (FPRP) for mobile ad hoc networks. Wirel. Netw..

[26] Rhee I., Warrier A., Min J., Xu L. (2009). DRAND: Distributed randomized TDMA scheduling for wireless ad hoc networks. IEEE Trans. Mob. Comput..

[27] Sato K., Sakata S. A distance-measurement-oriented distributed TDMA scheduling algorithm for sensor networks. Proceedings of the 2011 International Conference on Distributed Computing in Sensor Systems and Workshops (DCOSS).

[28] Sgora A., Vergados D.J., Vergados D.D. (2015). A survey of TDMA scheduling schemes in wireless multihop networks. ACM Comput. Surv. (CSUR).

[29] Bryan K.L., Ren T., DiPippo L., Henry T., Fay-Wolfe V. (2007). Towards Optimal TDMA Frame Size in Wireless Sensor Networks.

[30] Yao M., Lin C., Zhang P., Tian Y., Xu S. TDMA scheduling with maximum throughput and fair rate allocation in wireless sensor networks. Proceedings of the 2013 IEEE International Conference on Communications (ICC).

[31] Chang C., Chang C., Chen S., Tu S., Ho K. (2019). Optimisation-based time slot assignment and synchronisation for TDMA MAC in industrial wireless sensor network. IET Commun..

[32] Huang M., Liu A., Xiong N.N., Wang T., Vasilakos A.V. (2020). An effective service-oriented networking management architecture for 5G-enabled internet of things. Comput. Netw..

[33] Misra S., Sarkar S. (2015). Priority-based time-slot allocation in wireless body area networks during medical emergency situations: An evolutionary game-theoretic perspective. IEEE J. Biomed. Health Inf..

[34] Li M., Gu Z., Long Y., Shu X., Rong Q., Ma Z., Shao X. (2020). W-GPCR routing method for vehicular ad hoc networks. Sensors.

[35] Nasrallah A., Thyagaturu A.S., Alharbi Z., Wang C., Shao X., Reisslein M., ElBakoury H. (2019). Ultra-low latency (ULL) networks: The IEEE TSN and IETF DetNet standards and related 5G ULL research. IEEE Commun. Surv. Tutor..

[36] Soelistijanto B., Howarth M.P. (2014). Transfer reliability and congestion control strategies in opportunistic networks: A survey. IEEE Commun. Surv. Tutor..

[37] Surendran S., Prakash S. (2015). An ACO look-ahead approach to QoS enabled fault-tolerant routing in MANETs. China Commun..

[38] Akhtar N., Khan M.A., Ullah A., Javed M.Y. (2019). Congestion avoidance for smart devices by caching information in MANETs and IoT. IEEE Access.

[39] Wehrle K., Gunes M., Gross J. (2010). Modeling and Tools for Network Simulation.

[40] Calamoneri T. (2006). The L(h, k)-labelling problem: A survey and annotated bibliography. Comput. J..

[41] Alon N., Mohar B. (2002). The chromatic number of graph powers. Comb. Probab. Comput..

